# Sodium intake status in United States and potential reduction modeling: an NHANES 2007–2010 analysis

**DOI:** 10.1002/fsn3.248

**Published:** 2015-06-09

**Authors:** Sanjiv Agarwal, Victor L. Fulgoni, Lisa Spence, Priscilla Samuel

**Affiliations:** ^1^ NutriScience LLCEast NorritonPennsylvania; ^2^ Nutrition Impact LLCBattle CreekMichigan; ^3^ Tate & Lyle Ingredients Americas LLCDecaturIllinois

**Keywords:** NHANES, sodium intake, sodium reduction modeling, sodium reduction technology

## Abstract

Limiting dietary sodium intake has been a consistent dietary recommendation. Using NHANES 2007–2010 data, we estimated current sodium intake and modeled the potential impact of a new sodium reduction technology on sodium intake. NHANES 2007–2010 data were used to assess current sodium intake. The National Cancer Institute method was used for usual intake determination. Suggested sodium reductions using SODA‐LO ^®^ Salt Microspheres ranged from 20% to 30% in 953 foods and usual intakes were modeled by using various reduction factors and levels of market penetration. SAS 9.2, SUDAAN 11, and NHANES survey weights were used in all calculations with assessment across gender and age groups. Current (2007–2010) sodium intake (mg/day) exceeds recommendations across all age gender groups and has not changed during the last decade. However, sodium intake measured as a function of food intake (mg/g food) has decreased significantly during the last decade. Two food categories contribute about 2/3rd of total sodium intake: “Grain Products” and “Meat, Poultry, Fish & Mixtures”. Sodium reduction, with 100% market penetration of the new technology, was estimated to be 230–300 mg/day or 7–9% of intake depending upon age and gender group. Sodium reduction innovations like SODA‐LO ^®^ Salt Microspheres could contribute to meaningful reductions in sodium intake.

## Introduction

Dietary Guidelines for Americans 2010 (USDA 2010a) recommend a maximum dietary sodium intake of 2300 mg/day for the general population and 1500 mg/day for at‐risk groups, including African Americans, persons aged 51 years and above, and persons of any age with hypertension, diabetes, or chronic kidney disease (about half of the US population). The World Health Organization (WHO) (World Health Organization 2012) recommends adults consume less than 2000 mg of sodium, or 5 g of salt. Regardless of the recommendations, dietary sodium intake in the United States is well above that needed for physiological function and is greater than recommended. The scientific report of the Dietary Guidelines Advisory Committee 2015 (USDA 2015) has identified dietary sodium as a nutrient of public health concern due to over consumption is consistent with current sodium recommendations and sodium appears to be a key focus area for upcoming 2015 Dietary Guidelines. Excessive dietary sodium is a major contributor to hypertension which is a leading preventable risk factor for cardiovascular diseases (Sacks et al. 2001; Strazzullo et al. 2009; USDA 2010b, 2015; Aburto et al. 2013; Institute of Medicine 2013). Nearly 1 in 3 Americans or 68 million adults have hypertension, half of whom have uncontrolled hypertension (Gillespie et al. 2011), and in 2010, high blood pressure was estimated to be responsible for $156 billion in direct and indirect costs (Heidenreich et al. 2011).

Sodium is primarily consumed as sodium chloride (Institute of Medicine 2010). Very little sodium occurs naturally in foods, and the majority of sodium in the US diet is from sodium added during food processing as a food‐ingredient for flavor, processing aid, and for food safety purposes (Institute of Medicine 2010). Salt is also used by the food industry based on standards of identity for many products, and have functional effects in foods such as ice cream (minimizing water crystallization), baked goods (part of dough strengthening and leavening as bicarbonate). Packaged foods and restaurant foods contribute more than 75% of dietary sodium in the US diet with about 10% sodium consumption occurring naturally in foods and 5–10% from added salt during cooking or at a meal as discretionary salt (USDA 2010b).

In this study, we used the most recent, 2007–2010 data from the National Health and Nutrition Examination Survey (NHANES) to estimate the current sodium intake and modeled the potential impact on sodium intake via a new sodium reduction technology, SODA‐LO^®^ Salt Microspheres based upon potential usage levels in 953 foods. SODA‐LO^®^ is a salt‐reduction ingredient which can reduce sodium in certain applications through its technology that turns standard salt crystals into free‐flowing, hollow salt microspheres which efficiently delivers salt taste and functionality by maximizing surface area.

## Materials and Methods

### Study population

We used NHANES dietary intake data to assess sodium intake and its sources in the diets of US individuals. National Health and Nutrition Examination Survey is a large dietary survey conducted by the National Center for Health Statistics (NCHS) of the Center for Disease Control and Prevention (CDC) every year on a national representative sample of non‐institutionalized US individuals (USDA 2013). All participants or proxies provided written informed consent and the Research Ethics Review Board at the NCHS approved the survey protocol. Participants completed an in‐person 24 h dietary recall and health examination in a Mobile Examination Center. A second 24‐h dietary recall was collected via telephone 3–10 days after the Mobile Examination Center exam. Both 24‐h dietary recalls were collected using the United States Department of Agriculture's (USDA's) automated multiple‐pass method (AMPM). Detailed description of the survey design and the data collection procedures are available elsewhere (Center for Disease Control and Prevention and National Center for Health Statistics 2013). We combined data from NHANES 2007–2008 and 2009–2010 for the analyses. The combined sample included 17,387 participants (6090 for ages 2–18 years, 5908 for ages 19–50 years and 5389 for ages 51 years and above). Children under age 2 years and, pregnant and/or lactating females were excluded from the analyses.

### Estimation of sodium intake

The USDA food composition databases were used to determine the sodium derived from foods consumed by NHANES participants and reported in the 24‐h recall dietary interview. The USDA estimated the nutrient content of NHANES foods and recipes by linking the ingredients in survey foods and recipes to food composition data provided by the USDA's Nutrient Database for Standard Reference (SR). The SR‐Link file of the Food and Nutrient Database for Dietary Studies (FNDDS) versions 4.1 and 5.0 were used in conjunction with respective SR releases 22 and 24 to determine the nutrient content of NHANES 2007–2008 foods and NHANES 2009–2010 foods respectively (USDA 2009, 2010c, 2011; Ahuja et al. 2012). Sodium values, unadjusted for salt used in food preparation were used for all analyses. The usual intakes of sodium from all foods were determined using the National Cancer Institute (NCI) method for a single dietary component (Tooze et al. 2010). All analyses were adjusted for the complex survey design for NHANES and used appropriate sample weights. Covariates in the usual intake models included Dietary Reference Intake age groups, gender, day of the week of dietary recall (weekend/weekday) and interview sequence of the dietary recall (in person vs. via telephone).

### Estimation of food sources of sodium

Food and Nutrient Database for Dietary Studies 4.1 & 5.0 databases of USDA were used to define food groups for NHANES 2007–2008 and NHANES 2009–2010 dietary intake data, respectively (USDA 2009, 2010c, 2011; Ahuja et al. 2012). Data for over 7000 foods were collapsed into nine broad categories of FNDDS food groups. Sodium amount and the percent of total dietary sodium were computed for all FNDDS food groups.

### Sodium intake modeling analysis

We modeled 20–30% reduction in sodium content in 953 foods (17 foods in “Milk & Milk Products” for 20% reduction, 304 foods in “Meat, Poultry, Fish & Mixtures” for 20–25% reduction, 20 foods in “Egg” for 25% reduction, 30 foods in “Dry Beans, Peas, Other Legumes, Nuts & Seeds” for 25% reduction, 511 foods in “Grain Products” for 25% reduction, 35 foods in “Vegetable” for 20–30% reduction, and 36 foods in “Fats, Oils & Salad Dressings” for 25% reduction). Then by using sodium reduction levels noted above and a 10%, 50%, or 100% market penetration, we computed various scenarios for potential reduction in the usual intake of sodium. The individual reductions were computed for foods using the reduction factor and market penetration factor and were used to model usual intake after potential sodium reduction.

### Statistical methods

SAS 9.2 (SASs Institute, Inc.; Cary, NC, USA) and SUDAAN 11 (Research Triangle Institute, Research Triangle Park, NC, USA,) were used for all calculations. National Health and Nutrition Examination Survey weights, strata, and primary sampling units were used in all calculations to adjust for oversampling of certain groups, nonresponse by some selected sample persons, and to adjust for the complex sample design of NHANES to ensure national representative results. Data are presented as means ± standard errors (SE). *P* ≤ 0.01 was considered statistically significant.

## Results

**Figure 1:**
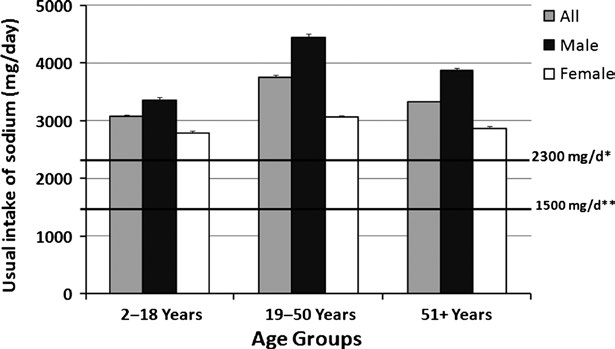
Usual intake of sodium by age and gender groups (Data from NHANES 2007–2010). Usual intakes from food were estimated by using the National Cancer Institute method. *Dietary Guidelines for Americans 2010 recommended level for general population; **Dietary Guidelines for Americans 2010 recommended level for at risk group.

Intake of sodium was age and gender dependent (Fig. 1). Usual intake of sodium among all adults (age 19–50 years) was 3756 ± 31 mg/day which was 22.2% higher than all children (age 2–18 years) and 13.0% higher than all older adults (age 51 years and above) with usual intakes of all children (age 2–18 years) at 3074 ± 32 mg/day and all older adults (age 51 years and above) at 3323 ± 21 mg/day. Usual intakes of sodium among males (children 3351 ± 53 mg/day, adults 4446 ± 55 mg/day and older adults 3866 ± 50 mg/day) were 20–45% higher than among females (children 2788 ± 38 mg/day, adults 3058 ± 33 mg/day and older adults 2869 ± 32 mg/day). A small proportion of population (22.1 ± 1.1% of children age 2–18 years, 8.1 ± 0.9% of adults age 19–50 years and 14.6 ± 0.8% of older adults age 51 and above) was consuming less than 2300 mg sodium per day. An even smaller proportion of the population (2.2 ± 0.3% of children age 2–18 years, 0.3 ± 0.1% of adults age 19–50 years and 0.8 ± 0.2% of older adults age 51 and above) was consuming less than 1500 mg of sodium per day.

**Figure 2:**
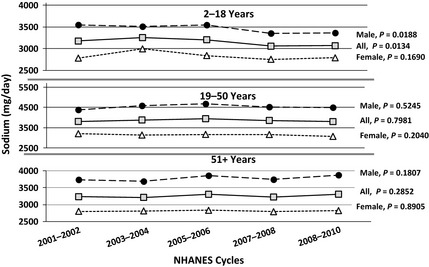
Trends in sodium intake by age and gender groups over 5 National Health and Nutrition Examination Survey (NHANES) cycles (Data from NHANES 2001–2010). Usual intakes from foods were estimated by National Cancer Institute method.

Figure 2 shows the trend in sodium intake (mg/day) over the past 5 NHANES cycles (NHANES 2001–2002 to NHANES 2009–2010) among the US population. Mean Intake of sodium in each NHANES cycle was higher than 2300 mg/day for all age and gender groups. Males in any age group consistently consumed more sodium than females in similar age groups in each NHANES cycle. Average intake of sodium (mg/day) during the past 5 NHANES cycles did not change significantly (*P* > 0.01) for any age or gender group. Average intake of sodium in NHANES 2001–2002 and NHANES 2009–2010 were 3174 ± 35 and 3067 ± 64 mg/day for children (age 2–18 years), 3812 ± 49 and 3805 ± 39 mg/day for adults (age 19–50 years), and 3233 ± 47 and 3306 ± 43 mg/day for older adults (age 51 years and above), respectively.

We also measured sodium intake as a function of energy intake (mg/kcal) and as a function of total food intake (mg/g food) in addition to absolute intake (mg/day) for all NHANES cycles. Table 1 shows trends in sodium intake by different measures over the last 5 NHANES cycles (2001–2010). During the past decade, while there was no change in sodium intake measured as mg/day, sodium intake measured as mg/kcal increased for some age/gender groups (for children regression coefficient (*β*) 0.0124, *P* = 0.003; for adults (age 19–50 years) *β*: 0.0219, *P* < 0.0001; and for male adults (age 19–50 years) *β*: 0.0284, *P* < 0.0001) and the changes were not significant for the remaining age/gender groups. Interestingly, sodium intake measured as a function of food intake (mg/g food) decreased significantly across all age and gender groups (*β*: −0.1114 to −0.1529, *P* < 0.0001) over the last 10 years (Table 1).

**Table 1:** Trends in sodium intake by age and gender groups over 5 NHANES cycles (Data from NHANES 2001–2010). Sodium intake was measured as absolute intake (mg/day) as a function of energy intake (mg/kcal) and as a function of food intake (mg/g food)

Age group	Gender group	Sodium intake trend					
		mg/day		mg/kcal		mg/g food	
		*β* 1	*P* 2	*β* 1	*P* 2	*β* 1	*P* 2
2–18 years	All	−41.12	0.0134	0.0124	0.0030	−0.1189	<0.0001
	Male	−52.54	0.0188	0.0122	0.0196	−0.1145	<0.0001
	Female	−22.03	0.1690	0.0132	0.0199	−0.1231	<0.0001
19–50 years	All	−3.93	0.7981	0.0219	<0.0001	−0.1332	<0.0001
	Male	16.67	0.5245	0.0284	<0.0001	−0.1142	<0.0001
	Female	−24.45	0.2040	0.0151	0.0416	−0.1529	<0.0001
51+ years	All	16.19	0.2852	0.0012	0.8025	−0.1227	<0.0001
	Male	34.27	0.1807	0.0055	0.5344	−0.1114	<0.0001
	Female	1.83	0.8905	−0.0025	0.6834	−0.1323	<0.0001

^1^ *β*, regression coefficient. ^2^ *P* < 0.01 significant. NHANES, National Health and Nutrition Examination Survey.

**Figure 3:**
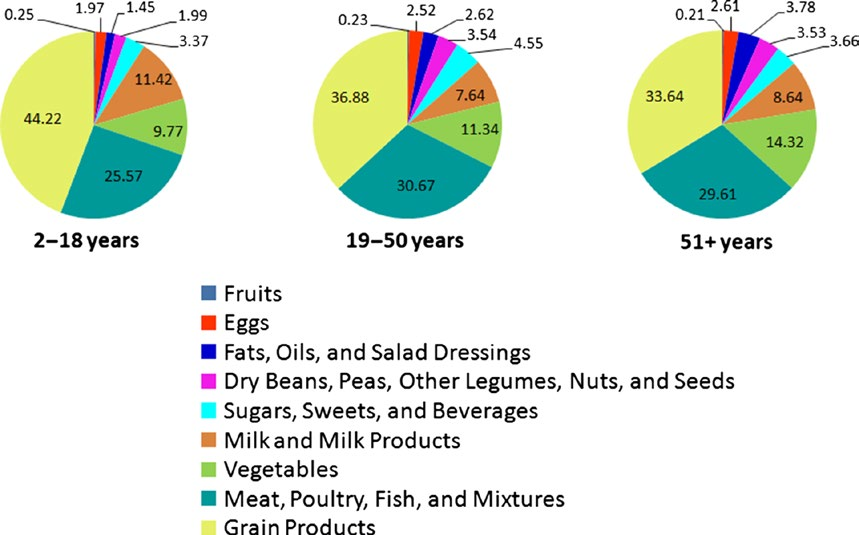
Dietary sodium contributions from nine FNDDS food groups by age groups in population subgroups (Data from NHANES 2007–2010). Data is presented as % of total dietary sodium.

Figure 3 shows the sodium contribution from 9 FNDDS defined food groups in the diets of US children, adults, and older adults (NHANES 2007–2010). No major overall age or gender related differences were noted. “Grain Products” and “Meat, Poultry, Fish & Mixtures” were the top two contributors of dietary sodium among children (44% and 26%, respectively), adults (37% and 31%, respectively) and older adults (34% and 30%, respectively) accounting for more than 60% of their total sodium intake. “Milk & Milk Products” and “Vegetables” were the next two highest contributors of dietary sodium among children (11% and 10%, respectively), adults (8% and 11%, respectively) and older adults (9% and 14%, respectively). These four food groups: “Milk & Milk Products”, “Meat, Poultry, Fish & Mixtures”, “Grain Products” and “Vegetables” were major contributors and provided more than 85% of sodium in the diet (children 91%, adults 87% and older adults 86%), and the remaining five food groups: “Eggs”, “Dry Beans, Peas, Other Legumes, Nuts & Seeds”, “Fruits”, “Fats, Oils & Salad Dressings”, and “Sugars, Sweets & Beverages” were minor contributors and provided less than 15% of sodium in the diet (children 9%, adults 13% and older adults 14%).

Table 2 shows the maximum achievable reductions (using the maximum reduction factor for each food and 100% market penetration) in sodium intake across all food categories using SODA‐LO^®^ technology to be 273 ± 7 mg (8.9 ± 0.1%), 301 ± 7 mg (7.9 ± 0.1%) and 231 ± 4 mg (7.1 ± 0.1%) in children (age 2–18 years), adults (age 19‐50 years) and older adults (age 51 years and above), respectively. A lower reduction factor and/or lower market penetration would provide lower reductions. Sodium reductions in the “Grain Products” category contributes more than 60%, and in the “Meat, Poultry, Fish & Mixtures” category contributes about 20% reduction of total sodium intake for any age group (Table 2).

**Table 2:** Potential sodium intake reduction with SODA‐LO^®^ Salt Microspheres (Salt Replacement Technology) by age groups (Data from NHANES 2007–2010) and FNDDS Food Groups. Potential reductions were modeled using 20–30% targeted reduction in sodium content in 953 foods with 100% market penetration

	Potential reduction (mg)	Current intake (mg)	Potential intake after reduction (mg)	% Reduction
All foods (953 foods; 20–30% targeted reduction in sodium content)				
2–18 years	273 ± 7	3053 ± 48	2780 ± 42	8.9 ± 0.1
19–50 years	301 ± 7	3813 ± 41	3512 ± 38	7.9 ± 0.1
51+ years	231 ± 4	3270 ± 43	3038 ± 42	7.1 ± 0.1
Milk and milk products (17 foods; 20% targeted reduction in sodium content)				
2–18 years	3 ± 1	319 ± 9	316 ± 8	0.2 ± 0.1
19–50 years	3.3 ± 0	283 ± 9	280 ± 9	0.4 ± 0.1
51+ years	5 ± 1	267 ± 10	262 ± 9	0.6 ± 0.1
Meat, poultry, fish and mixtures (304 foods; 20–25% targeted reduction in sodium content)				
2–18 years	61 ± 2	819 ± 20	759 ± 18	7.4 ± 0.2
19–50 years	71 ± 2	1205 ± 21	1134 ± 20	5.8 ± 0.2
51+ years	44 ± 2	1018 ± 23	974 ± 22	4.2 ± 0.2
Eggs (20 foods; 25% targeted reduction in sodium content)				
2–18 years	2 ± 0	60 ± 5	58 ± 4	1.1 ± 0.3
19–50 years	3 ± 0	96 ± 4	93 ± 4	1.6 ± 0.3
51+ years	3 ± 1	88 ± 4	85 ± 4	1.2 ± 0.2
Dry beans, peas, other legumes, nuts and seeds (30 foods; 25% targeted reduction in sodium content)				
2–18 years	4 ± 0	63 ± 5	59 ± 5	12.5 ± 0.7
19–50 years	5 ± 0	140 ± 8	135 ± 8	7.5 ± 0.5
51+ years	6 ± 0	117 ± 5	111 ± 5	9.7 ± 0.5
Grain products (511 foods; 25% targeted reduction in sodium content)				
2–18 years	179 ± 6	1347 ± 31	1168 ± 26	12.8 ± 0.2
19–50 years	187 ± 6	1396 ± 28	1210 ± 24	13.5 ± 0.3
51+ years	146 ± 3	1081 ± 24	935 ± 21	13.7 ± 0.23
Fruits (no targeted reduction in sodium content)				
2–18 years	0 ± 0	6 ± 0	6 ± 0	0.0 ± 0.0
19–50 years	0 ± 0	6 ± 1	6 ± 1	0.0 ± 0.0
51+ years	0 ± 0	6 ± 0	6 ± 0	0.0 ± 0.0
Vegetables (35 foods; 20–30% targeted reduction in sodium content)				
2–18 years	21 ± 1	298 ± 10	277 ± 10	8.3 ± 0.4
19–50 years	26 ± 1	439 ± 13	412 ± 13	6.4 ± 0.2
51+ years	19 ± 1	467 ± 12	448 ± 12	4.3 ± 0.2
Fats, oils and salad dressings (36 foods; 25% targeted reduction in sodium content)				
2–18 years	3 ± 0	48 ± 3	44 ± 3	17.1 ± 0.3
19–50 years	6 ± 0	99 ± 5	93 ± 5	13.9 ± 0.5
51+ years	9 ± 1	121 ± 6	112 ± 6	14.8 ± 0.4
Sugars, sweets and beverages (no targeted reduction in sodium content)				
2–18 years	0 ± 0	93 ± 4	93 ± 4	0.0 ± 0.0
19–50 years	0 ± 0	149 ± 3	149 ± 3	0.0 ± 0.0
51+ years	0 ± 0	106 ± 3	106 ± 3	0.0 ± 0.0

NHANES, National Health and Nutrition Examination Survey; FNDDS, Food and Nutrient Database for Dietary Studies.

## Discussion

The results show that sodium intake across all age and gender groups in 2007–2010 was significantly higher than the Dietary Guidelines for Americans 2010 (USDA 2010a) sodium recommendations and only a small proportion of the population was consuming less than the recommended amount. The current (2007–2010) intake of sodium was also higher than the American Heart Association 2010 recommendations (Lloyd‐Jones et al. 2010), WHO 2012 recommendations (World Health Organization 2012), the Institute of Medicine (IOM) defined Tolerable Upper Intake Level and Adequate Intake of sodium (Institute of Medicine 2005), and recommendations of other Scientific and Public Health Agencies and Organizations (USDA 2010b). High sodium intake is a global issue as demonstrated by average sodium intakes in other countries, including the UK, Japan, Brazil, and Spain (Brown et al. 2009).

Previous NHANES analysis (Gunn et al. 2010) also reported that in 2005–2006 the average intake of sodium for Americans age 2+ years was over 3400 mg/day and virtually all Americans consumed more sodium than needed. Cogswell et al. (2012) using the NHANES 2003–2008 data set, also reported that the vast majority of US adults consume more sodium than the recommended amount. Our data provide the most recent estimates for usual sodium intake among the US population in relation to current dietary recommendations. The present results also show that adults age 19–50 years consume more sodium than children age 2–18 years and older adults age 51 years and above, and males of any age group consume more sodium than females of the same age group. This observation may most likely be a function of higher food and calorie intake among adults and males. Current evidence suggests that consumption of excessive sodium is a risk factor for high blood pressure and hypertension (Sacks et al. 2001; Strazzullo et al. 2009; USDA 2010b, 2015; Aburto et al. 2013; Institute of Medicine 2013), and other consequent health outcomes, including coronary heart disease (CHD), stroke, and mortality. Our data show that despite consistent recommendations from government, scientific and public health agencies to limit sodium intake, sodium intake among the US population has not changed in the past decade and has remained consistently higher than recommendation for all age or gender groups. Cogswell et al. (2012) also reported that the average sodium intake in 2003–2008 did not significantly change from 1988 to 1994 published values across most population subgroups. However, in the present study, when we compared sodium intake as a function of food intake (mg/g food), we noted a significant decrease in sodium intake in all age gender groups over the last decade. This finding suggests a possibility of reduction in sodium content of foods with a consequent increase in food intake during 2001–2010. There has indeed been a significant increase in total food intake during the last decade (data not presented). This increase in food intake might have offset the decrease in sodium content of foods, resulting in no change in total sodium intake (mg/day) for the population. This finding emphasizes the need for innovative food technologies to help further reduce the sodium content of foods.

Sodium in foods is largely derived from salt added during processing as a flavor enhancer, as a processing aid and/or for food safety (Institute of Medicine 2010). Different foods contain different amounts of sodium. However, in addition to the sodium contents of foods, their consumption frequency is also an important factor contributing to daily sodium intake. In the US, the problem of excess sodium intake could be due to foods that may be moderate in sodium, but are frequently consumed (USDA 2010b). Our results show that “Grain Products” and “Meat, Poultry, Fish, & Mixtures” jointly provide about two‐third of the dietary sodium while “Dairy” and “Vegetables” contributed another one‐fifth of dietary sodium in 2007–2010. In a CDC study of NHANES 2005–2006 (Gunn et al. 2010), US adults consumed most sodium from grains (36.9% of daily sodium) and meat (26.9% of daily sodium). Similarly, NCI, using NHANES 2005–2006 data (National Cancer Institute 2013), also reported that the top 12 major food sources of sodium contributed 56% of dietary sodium among the US population age 2+ years and were mostly from “Grain Products” and “Meat, Poultry, Fish, & Mixtures”. These data suggest that “Grain Products” and “Meat, Poultry, Fish, & Mixtures” products are major sources of sodium in the diet and sodium reduction strategies aimed at these food groups compared to other food groups could have a bigger impact on dietary sodium reduction.

In spite of the consistent recommendations to limit sodium intake and evidence linking high sodium intake to hypertension, sodium intake in the US is high and has led to calls for population wide interventions to reduce sodium in US diets. In 2010 the IOM outlined primary and interim strategies for sodium intake reduction which included voluntary reduction of sodium content of foods (Institute of Medicine 2010). The strategy of the National Salt Reduction Initiative's, a partnership of more than 85 organizations and local and state health authorities, is to set feasible sodium reduction targets for 62 packaged food categories and 25 restaurant food categories (National Salt Reduction Initiative 2013). However, current sodium intake status (2007–2010 NHANES) and the sodium intake trend (2001–2010 NHANES) data presented above indicate that there has been no significant progress in the reduction of sodium intake. The food industry has tried to make small stepwise reduction in sodium content of foods. Our present dietary sodium modeling data, using SODA‐LO^®^ Salt Microspheres, a salt‐reduction ingredient at its potential usage level in 953 foods shows a 230–300 mg/day reduction in sodium intake which translates to about 7–9% of the current sodium intake among the US population. In a recently published report we demonstrated effectiveness of the SODA‐LO^®^ technology in reducing sodium intake in ethnic population subgroups (Fulgoni et al. 2014).

Reducing dietary sodium is an important target for public health improvement. Reduced sodium intake has been demonstrated to reduce blood pressure and is also associated with a reduced risk of stroke and fatal CHD in adults (He and MacGregor 2002; Aburto et al. 2013; He et al. 2014). A decrease of 2 mm Hg in diastolic blood pressure in the US population is estimated to result in 17% decrease in the prevalence of hypertension, a 6% decrease in risk of CHD, and a 15% decrease in risk of stroke, and could potentially prevent 67,000 CHD events and 34,000 stroke events every year in an analysis of data from Framingham Heart Study and NHANES II (Cook et al. 1995). Increased blood pressure is the leading modifiable risk factor for mortality and accounts for almost 13% of deaths globally (World Health Organization 2009). Several studies, using statistical modeling, have estimated the potential of overall health and health‐care cost benefits of dietary sodium reduction and have concluded that it is cost effective and its projected benefits are substantial (Asaria et al. 2007; Joffres et al. 2007; Dall et al. 2009; Danaei et al. 2009; Palar and Sturm 2009; Bibbins‐Domingo et al. 2010; Smith‐Spangler et al. 2010). It is estimated that dietary salt reduction could substantially reduce hypertension and related cardiovascular events and improve quality of life and provide economic benefit. Reducing dietary salt by 3 g (1200 mg sodium) per day was projected to reduce CHD, stroke and myocardial infarction, prevent deaths, and save $10–24 billion in health care costs annually using the CHD Policy Model, a computer simulation of heart disease among US adults (Bibbins‐Domingo et al. 2010). Interpolating the CHD Policy modeling data reported in Bibbins‐Domingo et al. (2010), our findings of a potential sodium reduction of 300 mg/day in adults in the age group 19–50 years, and assuming that the all things remain constant across the two studies, our findings would translate to a reduction of 0.45–0.88 mmHg in systolic blood pressure along with potential reductions of $3.0–5.3 billion in health care costs (Table 3).

**Table 3:** Projected estimates of health benefits and cost savings annually from sodium reduction. Data is presented as low estimate—high estimate

Benefit	Sodium reduction^a^ 1200 mg/day	Sodium reduction^a^ 400 mg/day	Sodium reduction 300 mg/day
SBP decrease^b^ (mmHg)	1.8–3.51	0.6–1.17	0.45–0.88
Total MI (reduction from expected events in thousands)	58–92	20–32	15–24
Stroke incidences (reduction from expected events in thousands)	37–59	13–20	9–15
CHD incidences (reduction from expected events in thousands)	66–110	22–37	17–28
Any cause death (reduction from expected events in thousands)	51–81	17–28	13–21
Health‐care cost reduction (billions)	$12.1–20.4	$4.1–7.0	$3.0–5.3

a Data from Bibbins‐Domingo et al. (2010).

b In total population excluding those with hypertension and age ≥65 years. SBP, Systolic Blood Pressure; MI, myocardial infarction; CHD, coronary heart disease.

SODA‐LO^®^ Salt Microspheres is a salt‐reduction ingredient technology that efficiently delivers salt taste and functionality by maximizing surface area due to its conversion of standard salt crystals into free‐flowing, hollow salt microspheres. Such a technology could modestly increase the cost of certain foods; however the potential health benefits, health improvement and reduced health care cost from dietary sodium reduction is expected to vastly out‐weigh the cost of technology. As salt intake is reduced, some people may adjust to food with less salt (He and MacGregor 2003). In such cases, there may be a concern that use of salt reduction technologies that do not alter flavor may potentially delay the consumer's palate from adjusting to lower sodium foods. However, changing consumer behavior is difficult and some attempts to lower dietary salt intake on an individual basis have largely proven to be ineffective (Hooper et al. 2004). Moreover, changing the palate may require a significant amount of time and in the interim technologies like SODA‐LO^®^ Salt Microspheres can provide immediate solution for sodium intake reduction.

A major strength of our study is the use of a large nationally representative population sample to assess the total usual intake of sodium with the NCI method. One of the limitations of our study was the cross‐sectional nature of NHANES data, which prevents directional conclusions or conclusions regarding causality.

## Conclusion

In conclusion, this study reports that current (2007–2010) sodium intake in the United States exceeds public health recommendations. Sodium reduction technologies such as SODA‐LO^®^ Salt Microspheres could potentially reduce dietary sodium intake by 7–9%.

## Conflict of Interest

S. Agarwal and V. L. Fulgoni, III are nutrition consultants and provide services to food industry. L. Spence is a full time employee of Tate & Lyle Ingredients America LLC. P. Samuel was a full time employee of Tate & Lyle LLC and has since left Tate & Lyle.
